# Multicenter, Randomized Split-Face Trial of a Crosslinked Hyaluronic Acid Fillers With Lidocaine for Nasolabial Fold Correction

**DOI:** 10.1093/asj/sjaf137

**Published:** 2025-08-01

**Authors:** Jeanine Downie, Michael Gold, John Joseph, Jeremy Green, Sabrina Fabi, David Bank, Joel L Cohen, Ava Shamban, Robert Weiss, Alice Krames-Juerss, Gary Monheit

## Abstract

**Background:**

Nasolabial folds (NLFs) are common age-related facial lines, often treated with dermal fillers. Princess FILLER Lidocaine (PFL; now saypha filler Lidocaine) and Juvéderm Ultra XC (JUXC) are both hyaluronic acid–based fillers used for this purpose.

**Objectives:**

The aim of the authors of this study is to evaluate the effectiveness and safety of PFL in reducing NLF severity compared with JUXC using a split-face study design.

**Methods:**

In this randomized, subject- and investigator-blinded multicenter study, patients with moderate-to-severe NLFs received PFL on one side of the face and JUXC on the other. Baseline NLF severity was assessed using the 5-point NLF-Severity Rating Scale (NLF-SRS). Follow-up assessments occurred at Weeks 12, 24, 36, and/or 48. The primary endpoint was the proportion of NLF-SRS responders at Week 24. Secondary endpoints included assessments by photographic reviewers and treating investigators, along with Global Aesthetic Improvement Scale (GAIS) ratings. Safety was monitored by adverse event reporting and patient diaries. FACE-Q questionnaires evaluated patient satisfaction. Repeat treatment was permitted at Week 36 or 48 if needed.

**Results:**

At Week 24, PFL demonstrated noninferiority to JUXC (82.2% vs 81.9% responders; difference 0.37%, *P* < .0001). Secondary assessments confirmed this finding. Adverse events occurred in 24.4% of patients post PFL, with most being mild to moderate. Serious treatment-emergent adverse events were rare (1.1%).

**Conclusions:**

PFL is a noninferior alternative to JUXC for treating moderate-to-severe NLFs, with comparable efficacy, safety, and patient satisfaction.

**Level of Evidence: 1 (Therapeutic):**

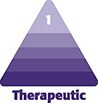

The use of hyaluronic acid (HA)-based soft tissue fillers to rejuvenate and beautify has witnessed a significant surge in demand, reflecting a growing societal acceptance and desire for procedures that enhance and rejuvenate one's appearance.^1^ Among the most commonly performed procedures, augmentation of the nasolabial folds (NLFs) has become a focal point for correction. NLFs tend to become more pronounced with age, exacerbated by the downward displacement of the subcutaneous superficial nasolabial fat compartment over the cutaneous insertion of the perioral musculature.^2,3^

For several decades, the approach to ameliorating NLFs has involved the use of various filler substances, with a long history of both permanent and nonpermanent injectables being implemented.^4^ Over the last 2 decades, HA-based soft tissue fillers have risen to the forefront, largely because of their impressive safety profile, high patient satisfaction, and tangible effectiveness.^5-7^ By providing immediate volume under the skin within the superficial or deep subcutaneous fat, HA-based soft tissue fillers are able to smooth out folds and wrinkles. Because the demand for aesthetic procedures escalates, the market has responded with an influx of HA-based soft tissue fillers. The assortment available to clinicians and patients varies not only in HA concentration and volume but also in the proprietary techniques employed for cross-linking the HA molecules. Cross-linking, connecting the individual polymer chains into a network, is a critical factor in determining the filler's durability, viscosity, and tissue integration.^8^ Different cross-linking agents and processes result in a spectrum of products tailored to specific treatment objectives and preferences. This diversity allows for personalized treatment plans, aligning the choice of filler with the desired aesthetic outcome, longevity of effect, and individual patient anatomy and needs.^9,10^ Princess FILLER Lidocaine (PFL) approved for use in the European Union since 2016 (now saypha filler Lidocaine; submitted for the US approval in 2024, Croma Pharma GmbH, Leobendorf, Austria) is a sterile, biodegradable, homogenized, and isotonic HA gel that has been crosslinked using 1,4-butanediol diglycidyl ether, a process that creates covalent bonds between HA molecules to form a stable 3-dimensional matrix. Further, 0.3% w/w lidocaine hydrochloride is added, which serves to diminish pain or discomfort during and after injection. Its rheological properties make it a suitable substance for the amelioration of the NLFs and has been shown in previous investigations to create safe and satisfying results.^11^ This current clinical investigation is designed to further evaluate the performance of PFL by juxtaposing it with another industry-standard HA filler, Juvéderm Ultra XC (JUXC). Through a randomized, controlled, multicenter, and split-face methodology, the study affords a direct comparison between the 2 products, thereby enabling a robust assessment of their relative effectiveness and safety in the correction of NLFs.

## METHODS

### Study Design

**Figure 1.**
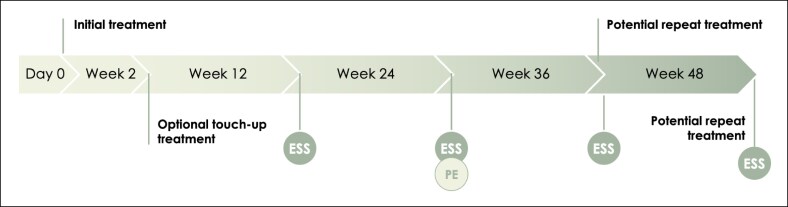
Treatment and evaluation timeline. This diagram outlines the treatment schedule and evaluation points across a 48-week period. Initial treatment occurs at Day 0, followed by an optional touch-up treatment at Week 2. Primary endpoint evaluation is performed at Week 24. Secondary effectiveness endpoints, subject satisfaction, and safety assessment have been conducted at Weeks 12, 24, 36, and 48. Potential repeat treatments may be administered at Weeks 36 and 48 based on patient response and clinical assessment.

This was a randomized, patient- and evaluating investigator–blinded, active treatment controlled, noninferiority, multicenter, paired (split-face) comparison clinical investigation of PFL vs JUXC in the treatment of moderate-to-severe NLFs, conducted at 10 sites in the United States by 10 investigators. The clinical investigation plan (CIP), CIP amendments, informed consent forms, and any other appropriate study-related documents were reviewed and approved by a central IRB (Advarra Institutional Review Board, Columbia, MD) for each study center and the study was registered at ClinicalTrials.gov (US National Library of Medicine, Washington, DC), NCT03990883. The investigation was performed in accordance with the International Standards Organization ISO14155, the principles of the Declaration of Helsinki, and the applicable sections of the respective national laws. The purpose of the investigation was to evaluate the effectiveness and safety of PFL in the correction of moderate-to-severe NLFs compared with JUXC. Safety and effectiveness assessments were carried out at Weeks 12, 24, 36, and/or 48. Additional safety evaluations were scheduled for Week 2, 4, and 6, with the latter being conditional on whether a touch-up was administered at Week 2. A repeat treatment was an option at Week 36 or 48. In instances where a repeat treatment was administered, subsequent safety visits were then slated 4 and 12 weeks later. In the study, some participants did not receive repeat treatment evaluations at Week 36 and/or Week 48 because of a pause in repeat treatments starting May 15, 2020. This was to enhance the monitoring and reporting of potential adverse events (AEs), particularly ophthalmological ones. The pause ended on June 21, 2021, and a new follow-up (Visit 7c) was introduced for evaluations and potential repeat treatments. Repeat treatments were followed by safety checks 4 and 12 weeks later (Figure 1).

### Study Cohort

Each treated patient received both the devices under investigation, PFL, and the comparator, JUXC. Eligible participants for the clinical investigation were required to be 22 years or older, of any gender, as determined at the initial screening visit. They should have presented with 2 clearly visible and roughly symmetrical NLFs of moderate or severe classification, consequently both scoring either 2 or 3 on the 5-point NLF Severity Rating Scale (NLF-SRS), as evaluated by the treating investigator and confirmed by the blinded evaluating investigator.

Female participants of childbearing potential were mandated to produce a negative urine pregnancy test at the time of injection visits and were required to commit to using a reliable form of birth control for the entirety of the study period. Candidates were also expected to have healthy skin in the nasolabial region, without any diseases that could affect the assessment. Additionally, participants needed to agree not to undergo any aesthetic or surgical procedures in the treatment area, which included refraining from botulinum toxin injections, although treatments for the glabellar or forehead area were permissible. Lastly, they must have demonstrated a clear understanding of the study's purpose and procedures, with written informed consent provided before participating in the clinical investigation.

Individuals were excluded from the clinical investigation if they were pregnant, lactating, or planning pregnancy; had allergies to HA, lidocaine, or gram-positive bacterial proteins; possessed a tendency for keloids, hypertrophic scars, or pigmentation disorders; were HIV-positive; had infections, inflammatory conditions, cancerous lesions in the treatment area; suffered recurrent herpes simplex; had autoimmune or connective tissue diseases; were undergoing immunomodulating therapy; had uncontrolled diabetes or systemic diseases; had undergone certain facial procedures or planned to; were using anticoagulant medication close to injection time; required dental surgery around the time of injection; or had any condition deemed unsuitable by the investigator. Also excluded were those previously enrolled in this study, participating in other clinical investigations, or patients dependent on investigators or the clinical site. Lastly, unsatisfactory visual examination results also led to exclusion. The assignment of fillers to the respective NLF sides was determined through a randomization process through an interactive web response system during the baseline visit. Participants in Group A received PFL for the left NLF and JUXC for the right NLF. Conversely, those in Group B were administered JUXC on the left NLF and PFL on the right NLF. The treating investigator administering the treatment was aware of the device injected (unblinded treating investigator). The independent blinded evaluating investigator was not aware of the device injected. Patients were also blinded to the treatment administered (they were blindfolded during the administration of devices). Based on photographs, NLF severity was assessed by 3 independent blinded photographic reviewers at the end of the clinical investigation.

### Treatment

The study device injections were administered into the mid-to-deep dermis. After an initial injection of a small quantity to allow the lidocaine to take effect for ∼3 s, investigators were permitted to use different injection techniques based on their clinical judgment and the anatomical characteristics of the defect under correction. Techniques such as the retrograde technique—involving injection while slowly withdrawing the needle after full insertion—and the fan technique—injecting in multiple directions from a single-entry point to cover a broader area—were allowed. This flexible approach aimed to reflect typical clinical practice. Injection volumes were tailored to achieve optimal correction during the initial session and were recorded in the electronic case report form. All injections followed the respective device's Instructions for Use, with a maximum of 10 mL per session and 20 mL per patient per year. For JUXC, dosing was further limited to 20 mL per 60 kg of body mass annually. PFL was supplied in 1.0 mL syringes with two 27 G needles, and JUXC in 1.0 mL syringes with 30 G needles, both labeled per regulatory standards. A touch-up treatment was permitted at Week 2 if optimal aesthetic correction had not been achieved. Repeat treatment was allowed at Week 36 or beyond if the patient’s NLFs had returned to the baseline severity level per the investigator's assessment. Repeat treatment was performed with PFL in both NLFs.

### Effectiveness Assessments

**Figure 2.**
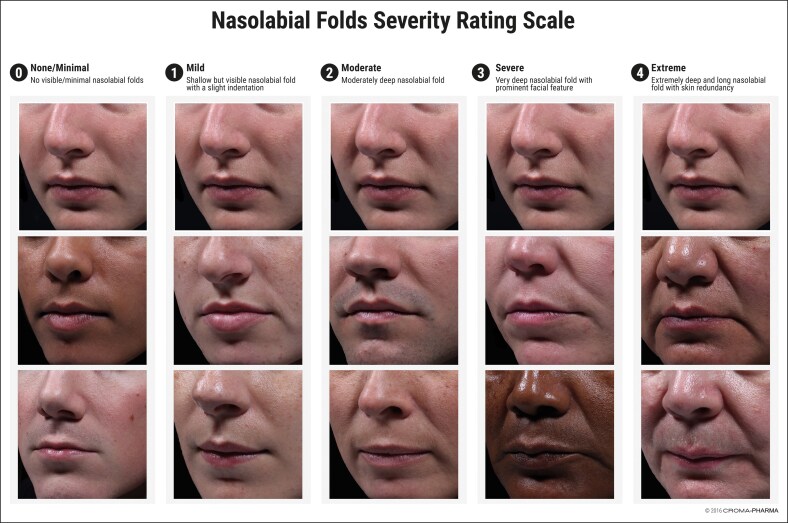
Nasolabial Folds Severity Rating Scale (NLF-SRS). NLF-SRS is a validated 5-point rating scale ranging from Grade 0 (none/minimal: no visible or minimal wrinkles) to Grade 4 (extreme: extremely deep and long NLF with skin redundancy). After enrollment, live assessments using the NLF-SRS were performed by the treating investigator and the independent, blinded, evaluating investigator (separate assessments) at the time points indicated in the schedule of assessments. Photographic assessments using the NLF-SRS were performed at the end of the clinical investigation by 3 independent blinded photographic reviewers. The left and the right NLFs were graded separately. Reproduced with permission from Croma-Pharma GmbH (Leobendorf, Austria).

The effectiveness of the treatment was measured using the NLF-SRS, a validated 5-point scale ranging from 0 (none/minimal) to 4 (extreme) (Figure 2). The primary effectiveness endpoint was the proportion of NLF-SRS responders at Week 24 after initial treatment with PFL compared with JUXC based on the independent blinded evaluating investigator live assessment, with response defined as at least 1-point improvement on the scale. Both the treating investigator and an independent blinded evaluating investigator assessed each patient's left and right NLFs live and separately used the NLF-SRS at various time points. Additionally, photographic reviews by 3 independent blinded reviewers were conducted at the study's end. Another measure was the Global Aesthetic Improvement Scale (GAIS), a 5-point scale assessing overall aesthetic improvement from pretreatment (Table 1). This evaluation was independently performed by both the patient and the blinded evaluating investigator for each side of the face at specified times. The GAIS grades ranged from 1 (very much improved) to 5 (worse), with intermediate grades indicating varying degrees of improvement or lack thereof. NLF-SRS and GAIS were assessed at baseline and Weeks 12, 24, 36, and if no retreatment was performed at Week 36 also at Week 48. The study used 2 FACE-Q questionnaires (FACE-Q is a US-registered trademark of Memorial Sloan-Kettering Cancer Center, 1275 York Avenue, New York, NY 10065. © 2013 Memorial Sloan-Kettering Cancer Center, Memorial Hospital for Cancer and Allied Diseases, Sloan-Kettering Institute for Cancer Research, Anne Klassen, and Stefan Cano. All rights reserved.) to measure patient satisfaction and conduct an appraisal of their NLFs post procedure. The Subject Satisfaction with Outcome Questionnaire comprised 6 questions, each answerable on a scale from 1 (definitely disagree) to 4 (definitely agree), leading to a possible score range of 6 to 24. Higher scores indicated greater satisfaction. These scores were then converted into a Rasch-transformed score ranging from 0 (worst) to 100 (best).

**Table 1.** Table Showing the Grades and Their Respective Definition of Global Aesthetic Improvement Scale

Grade		Definition
1	Very much improved	Optimal aesthetic result for the implant in this patient
2	Much improved	Marked improvement in appearance from initial condition, but not completely optimal for this patient. A touch-up would slightly improve the result
3	Improved	Obvious improvement in appearance from the clinical condition, but a touch-up or retreatment is indicated
4	No change	The appearance is essentially the same as the original condition
5	Worse	The appearance is worse than the original condition

Similarly, the Subject Appearance Appraisal of Nasolabial Folds Questionnaire contained 5 questions with a scoring range from 1 (not at all) to 4 (extremely), summing up to a score between 5 and 20. Here too, higher scores denoted a more favorable outcome, and these were converted into a Rasch-transformed score from 0 (worst) to 100 (best). Patients performed these assessments separately for both the left and the right sides of their face at designated times in the study. For each patient, the total volume injected was recorded separately for each treatment (initial, touch-up, and repeat treatments).

### Safety Assessments

Throughout the study, the occurrence of AEs, serious AEs, adverse device events, and device deficiencies (DDs) were meticulously documented. Additionally, the use of any concurrent medications by participants was also recorded. Furthermore, patients documented injection-site reactions (ISRs) by paper diary.

### Statistical Analysis

In the study, continuous variables were analyzed by noting the number of observations (*n*), mean, standard deviation, median, minimum, and maximum values. Categorical variables were summarized based on the number of observations (*n*), frequency, and percentage of patients involved. The precision of these summary statistics is detailed in the Statistical Analysis Plan, and all relevant data from patients in the database were included in listings. For the primary analysis, patients missing NLF-SRS grades at baseline or Week 24 were classified as nonresponders. An NLF-SRS response was individually determined for each NLF, with a patient considered a responder if there was at least a 1-grade improvement from baseline in that specific NLF.

The primary effectiveness endpoint involved testing the hypothesis: H0 (null hypothesis) stated that the difference in response rates between PFL (pA) and JUXC (pB) was less than or equal to the noninferiority margin d0, set at −10%. A negative value in the difference (pA-pB) indicated a lower response rate for PFL compared with JUXC. Noninferiority was assessed using a 1-sided McNemar type test at an alpha level of 0.025 with a delta of 10%. PFL would be considered noninferior if the lower limit of the 2-sided 95% CI (equivalent to the 1-sided 97.5% CI) for the difference between proportions was >−10%.

The primary effectiveness endpoint was assessed using the 1-sided 95% CI of the difference between paired response rates (pA−pB), where pA represents the response rate for PFL and pB represents that for JUXC. Noninferiority was concluded if the lower bound of the 95% CI did not exceed the prespecified noninferiority margin of −10%. Sample size estimation was based on an assumed response rate at Week 24 of 88% for JUXC and 87% for PFL. Assuming the 2 NLFs of each patient respond independently, 21.9% of patients were expected to respond on only 1 side and 76.5% on both sides. This assumption was considered conservative, because some degree of within-patient correlation was expected. Based on these parameters, a sample of 222 patients was required to achieve 90% power, calculated using nQuery Advisor 7.0 with 1600 simulations applying the Newcombe–Wilson score method for CI estimation.

## RESULTS

### Participant Demographics and Baseline Characteristics

**Figure 3.**
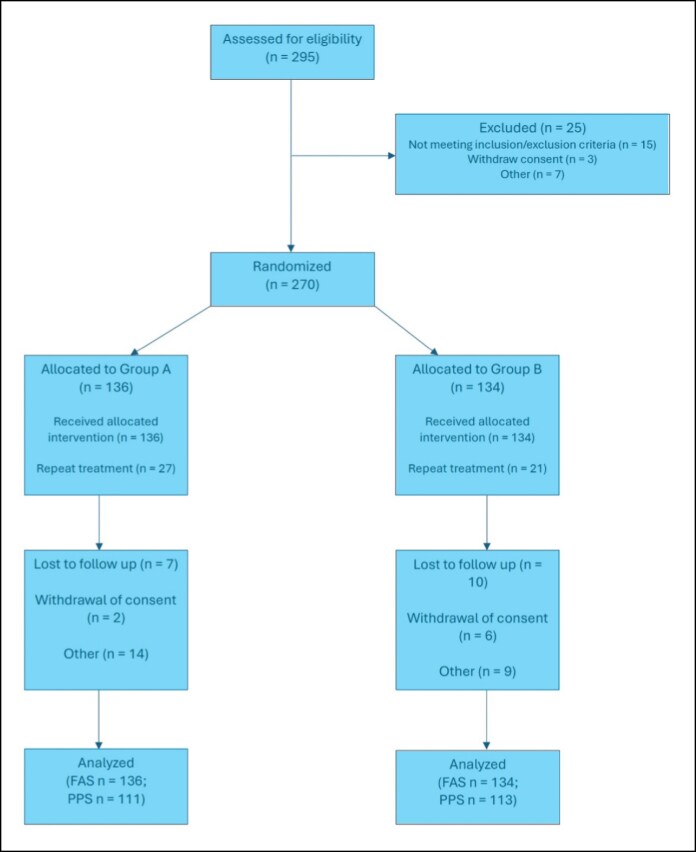
CONSORT flow diagram.^12^

Out of 295 patients initially screened for the study, 25 were screening failures. Eventually, 270 patients were suitable and were randomized: 136 to Group A and 134 to Group B, with all undergoing treatment. A total of 48 patients, 27 from Group A and 21 from Group B, underwent a repeat treatment using PFL. By the study's conclusion, 222 patients (82.2%) completed the investigation, comprising 113 (83.1%) from Group A and 109 (81.3%) from Group B. A total of 48 patients (17.8%) withdrew early, including 23 (16.9%) from Group A and 25 (18.7%) from Group B (Figure 3).

Overall, the patients had an average age of 55.4 ± 9.4 years (range, 30-86 years) and an average body weight of 70.92 ± 14.7 kg at screening. A majority were female (264 patients, 97.8%) and of nonchildbearing potential (183 out of 264 female patients, 67.8%). The predominant Fitzpatrick skin types was Type II (78 patients, 28.9%), Type III (81 patients, 30.0%), and Type IV (63 patients, 23.3%) at baseline. A total of 99 patients (36.7%) had Fitzpatrick skin Type IV, V, or VI, with 36 patients (13.3%) having Type V or VI at baseline. The group consisted of individuals from diverse ethnic backgrounds, with 83.7% being White (226 individuals), 10.7% Black or African American (29 individuals), 3.7% American Indian or Alaska Native (10 individuals), and 0.7% for Asian (2 individuals). Additionally, there was 1 participant (0.4%) who identified as Native Hawaiian or Other Pacific Islander. Of the 270 NLFs treated with PFL, 107 (39.6%) were moderate and 163 (60.4%) severe at baseline. Of the 270 NLFs treated with JUXC, 109 (40.4%) were moderate and 161 (59.6%) severe at baseline.

### Treatment Administration

For the initial treatment, PFL had a mean injection volume of 1.24 ± 0.58 mL, whereas JUXC exhibited a mean injection volume of 1.03 mL ± 0.44 mL. With regard to touch-up treatments, 147 NLFs treated with PFL required a touch-up, with a mean injection volume of 0.43 ± 0.23 mL. In contrast, 152 individuals using JUXC required a touch-up, with a mean injection volume of 0.39 ± 0.20 mL. As for repeat treatments, at Week 36, 13 NLFs with PFL received an additional treatment with a mean volume of 0.62 ± 0.28 mL, whereas 13 NLFs using JUXC received a mean volume of 0.58 ± 0.21 mL. At Week 48, 1 NLF received a treatment with PFL (0.70 mL) and 1 with JUXC (0.80 mL). Lastly, at Visit 7c, 34 individuals with PFL received a repeat treatment with a mean volume of 0.83 ± 0.39 mL, whereas for JUXC, 34 individuals received a repeat treatment with a mean volume of 0.86 ± 0.39 mL.

### Effectiveness

#### Nasolabial Folds Severity Rating Scale (NLF-SRS)

**Figure 4.**
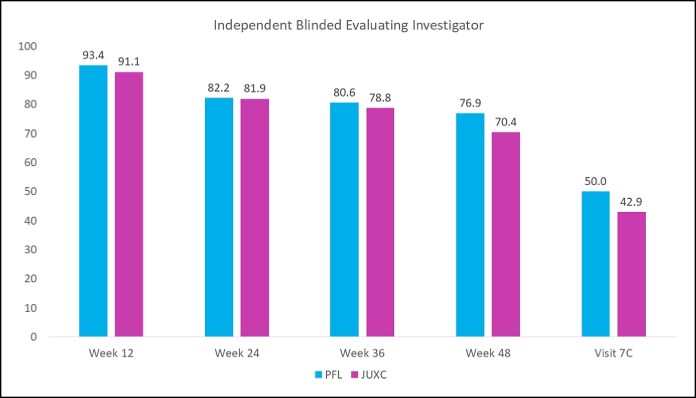
Bar graph showing the percentage of responders, defined as having at least 1 grade improvement over baseline on the 5-point Nasolabial Folds Severity Rating Scale, as assessed by the independent blinded evaluating investigator.

**Figure 5.**
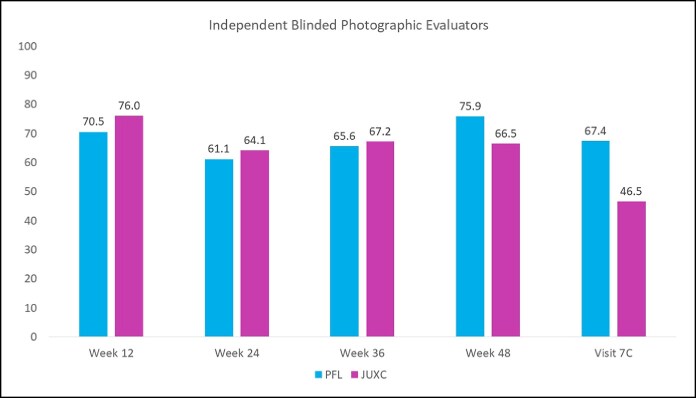
Bar graph showing the percentage of responders, defined as having at least 1 grade improvement over baseline on the 5-point Nasolabial Folds Severity Rating Scale, as assessed by independent, blinded photographic evaluators.

Based on the independent blinded evaluating investigator live assessment, both PFL and JUXC showed similar response rates in reducing NLF-SRS symptoms within the FAS after the initial treatment at Week 24, with 82.2% of patients for PFL and 81.9% for JUXC. PFL demonstrated noninferiority to JUXC, because the difference in responder rates was only 0.37%, and the lower boundary of the 95% CI (−2.96) exceeded the noninferiority margin of −10% (*P* < .0001). Furthermore, PFL resulted in a slightly higher percentage of patients achieving a 2-point (34.1% vs 30.4%) or 3-point (8.5% vs 6.7%) reduction from baseline on the NLF-SRS compared with JUXC. When independent photographic reviewers analyzed the NLF-SRS assessments for Week 24, they observed a lower percentage of responders following each treatment. Specifically, 61.1% of patients treated with PFL and 64.1% of those treated with JUXC were responders. The noninferiority of PFL to JUXC remained supported. The difference of proportions was −2.96 (−9.99, 4.07] (*P* = .025) exceeding the noninferiority margin of −10%. Based on the assessment of the treating investigator at Week 24, the response rates were 83.3% for patients treated with PFL and 83.7% for patients treated with JUXC. The noninferiority of PFL to JUXC was supported by this analysis (difference of proportions was −0.37 (−3.85, 3.11); *P*-value was <.0001). The injection techniques (fanning and retrograde) showed a significant difference only at the 12-week follow-up for PFL. The responder rate was 95.4 for the retrograde technique and 87.3% for the fanning technique (*P* = .0253). At the subsequent follow-ups (Weeks 24, 36, and 48), the differences in responder rates between the 2 techniques were not statistically significant. No significant difference was found for the injection technique when injecting JUXC at any time point. All effectiveness analyses were repeated in the PPS, which confirmed the results obtained for the FAS. NLF-SRS values over time are given in Figures 3-5 (Table 2).

**Table 2.** Summary of the Proportion of Nasolabial Folds Severity Rating Scale Responders at Weeks 12, 36, 48, and Visit 7C After Initial Treatment With PFL Compared With JUXC

	Independent blinded evaluating investigator live assessment		Independent blinded photographic reviewer		Treating investigator	
	PFL	JUXC	PFL	JUXC	PFL	JUXC
Week 12	93.4	91.1	70.5	76	95	95
Week 24	82.2	81.9	61.1	64.1	83.3	83.7
Week 36	80.6	78.8	65.6	67.2	82.9	80.3
Week 48	76.9	70.4	75.9	66.5	75.8	66.8
Visit 7C	50	42.9	67.4	46.5	40.5	31

JUXC, Juvéderm Ultra XC; PFL, Princess FILLER Lidocaine.

#### Global Aesthetic Improvement Scale

Based on the assessment of the independent blinded evaluating investigator, similar proportions of patients in each treatment group were classified as GAIS responders at Week 24 following initial treatment, with 93.2% of patients for PFL and 92.8% of patients for JUXC. The difference in the proportion of GAIS responders between the 2 treatments (PFL vs JUXC) was found to be 0.40% (−2.65, 3.45).

Based on the patient's assessment, the response rate was consistent between the 2 treatments, with 91.6% for each treatment (Table 3).

**Table 3.** Summary of the Proportion of GAIS Responders at Week 12, 24, 36, 48, and Visit 7C After Initial Treatment With PFL Compared With JUXC

	Independent blinded evaluating investigator live assessment		Patient	
	PFL	JUXC	PFL	JUXC
Week 12	97.3	96.5	98.4	95.7
Week 24	93.2	92.8	91.6	91.6
Week 36	92.4	89.4	92.1	91.1
Week 48	84.9	84.9	88.2	84.8
Visit 7C	62.5	57.5	66.7	66.7

JUXC, Juvéderm Ultra XC; PFL, Princess FILLER Lidocaine.

#### FACE-Q

FACE-Q Subject Satisfaction was comparable for both products at various time points. Specifically, at Week 12, the mean-transformed score was 74.1 ± 23.89 for PFL and 73.1 ± 23.69 for JUXC. At Week 24, it was 70.0 ± 24.97 for PFL and 68.3 ± 25.46 for JUXC. Moving to Week 36, the scores were 69.0 ± 22.88 for PFL and 67.8 ± 23.78 for JUXC. At Week 48, they were 66.4 ± 23.72 for PFL and 66.0 ± 22.70 for JUXC. For the patients followed up at Visit 7c, the scores were 51.8 ± 26.91 for PFL and 48.8 ± 25.88 for JUXC.

Furthermore, as described in the Supplemental Table 1, available online at https://doi.org/10.1093/asj/sjaf137, 90.0% of patients treated with PFL were pleased with the result at Week 12, 83.3% at Week 24, 69.3% at Week 36, and 71.5% at Week 48 (sum of 2 categories “somewhat agree” + “definitely agree”).

The change from baseline for FACE-Q Subject Appraisal of NLFs score was comparable for both fillers at different time points. At Week 12, the mean scores were 46.3 ± 29.96 for PFL and 45.8 ± 29.80 for JUXC. At Week 24, they were 41.2 ± 28.78 for PFL and 39.3 ± 28.69 for JUXC. Moving to Week 36, the scores were 34.4 ± 30.54 for PFL and 31.9 ± 31.33 for JUXC. At Week 48, they were 30.5 ± 30.35 for PFL and 30.2 ± 30.50 for JUXC. For the patients followed up at Visit 7c, the scores were 15.0 ± 27.16 for PFL and 15.1 ± 26.11 for JUXC.

### Adverse Events

Treatment-emergent adverse events (TEAEs), including device effects, were reported in 67 (24.8%) patients following initial or touch-up treatments: 66 (24.4%) after treatment with PFL and 63 (23.3%) after treatment with JUXC. Among these, 22 (8.1%) patients experienced TEAEs related to the study procedure, with 21 (7.8%) after treatment with the PFL group and 21 (7.8%) after the JUXC group. Additionally, 16 (5.9%) patients reported TEAEs related to the study device, with 15 (5.6%) after treatment with PFL and 14 (5.2%) after treatment with the JUXC group. Most TEAEs were of mild or moderate severity.

After initial/touch-up treatments with either PFL or JUXC, the maximum duration of study device–related TEAEs was approximately evenly spread across 1 to 14 days in both groups; 3 patients and 2 patients, respectively, had study device-related TEAEs that lasted longer than 14 days. Serious TEAEs were reported in 3 (1.1%) patients following initial or touch-up treatments with both PFL and JUXC. Treatment-emergent AEs (TEADEs) were observed in 26 patients (9.6%) following their initial or touch-up procedures using PFL, and in 24 patients (8.9%) after similar treatments with JUXC. The most frequent TEADEs, affecting >2 patients in the total group, included headaches in 6 patients (2.2%), along with swelling and contusions in 4 patients (1.5%) each. Additionally, discomfort, eyelid margin crusting, and injection-site erythema were each reported in 3 patients (1.1%). One patient had a serious treatment-emergent adverse device effect (TEADE), which was also considered an unanticipated serious TEADE. No TEAE or TEADE resulted in death. TEAEs of special interest were reported in 3 (1.1%) patients after initial or touch-up treatments with both PFL and JUXC. Furthermore, a TEAE or TEADE leading to study withdrawal was reported for 1 (0.4%) patient following initial or touch-up treatments with PFL and JUXC. After initial treatments, 243 patients (95.3%) experienced ISRs with PFL, and 238 patients (92.6%) with JUXC. Most ISRs were mild (91 patients, 35.7%) or moderate (PFL: 117 patients, 45.9%; JUXC: 115 patients, 44.7%). Severe ISRs were reported by 35 patients (13.7%) using PFL and 32 patients (12.5%) with JUXC. Common ISRs, experienced by over 50% of patients, included firmness (PFL: 205 patients, 80.4%; JUXC: 199 patients, 77.4%), swelling, lumps/bumps, tenderness, redness, and bruising. Over 5% of patients reported severe bruising, lumps/bumps, and firmness (Table 4).

**Table 4.** Overall Summary of Treatment-Emergent Adverse Events After Initial/Touch-up Treatment

Category	PFL (*n* = 270)	JUXC (*n* = 270)	Total (*n* = 270)
	*n* (%)	*n* (%)	*n* (%)
TEAEs (all TEAEs including device effects)	66 (24.4)	63 (23.3)	67 (24.8)
Study device-related	15 (5.6)	14 (5.2)	16 (5.9)
Study procedure-related	21 (7.8)	21 (7.8)	22 (8.1)
TEAEs maximum relationship (including study device and study procedure)			
Related	26 (9.6)	24 (8.9)	27 (10.0)
Not related	40 (14.8)	39 (14.4)	40 (14.8)
Serious TEAEs	3 (1.1)	3 (1.1)	3 (1.1)
TEAEs maximum severity			
Severe	5 (1.9)	5 (1.9)	5 (1.9)
Moderate	18 (6.7)	17 (6.3)	18 (6.7)
Mild	43 (15.9)	41 (15.2)	44 (16.3)
TEAEs leading to study withdrawal	1 (0.4)	1 (0.4)	1 (0.4)
TEAEs leading to patient death	0	0	0
TEAEs of special interest	3 (1.1)	3 (1.1)	3 (1.1)
TEADEs	26 (9.6)	24 (8.9)	27 (10.0)
Study device-related	15 (5.6)	14 (5.2)	16 (5.9)
Study procedure-related	21 (7.8)	21 (7.8)	22 (8.1)
TEADEs maximum relationship (including study device and study procedure)			
Related	26 (9.6)	24 (8.9)	27 (10.0)
Serious TEADEs	1 (0.4)	1 (0.4)	1 (0.4)
Unanticipated serious TEADEs	1 (0.4)	1 (0.4)	1 (0.4)
Anticipated serious TEADEs	0	0	0
TEADEs maximum severity			
Severe	3 (1.1)	3 (1.1)	3 (1.1)
Moderate	3 (1.1)	2 (0.7)	3 (1.1)
Mild	20 (7.4)	19 (7.0)	21 (7.8)
TEADEs leading to study withdrawal	1 (0.4)	1 (0.4)	1 (0.4)
TEADEs leading to patient death	0	0	0

ADE, adverse device effect; AE, adverse event; ASADE, anticipated serious adverse device effect; eCRF, electronic case report form; IMD, investigational medical device; TEADE, treatment-emergent adverse device effect; TEAE, treatment-emergent adverse event; USADE, unanticipated serious adverse device effect.

## DISCUSSION

**Figure 6.**
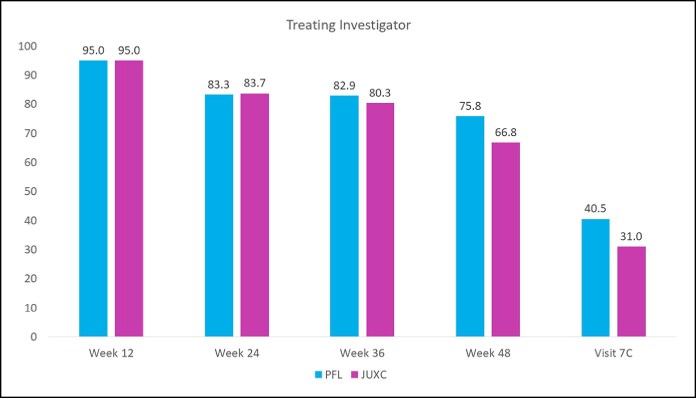
Bar graph showing the percentage of responders, defined as having at least 1 grade improvement over baseline on the 5-point Nasolabial Folds Severity Rating Scale, as assessed by the treating investigator.

This study was a randomized, patient- and investigator-blinded, noninferiority trial comparing PFL and JUXC in treating moderate-to-severe NLFs across 10 US centers. It involved patients aged 22 years and above, utilizing a split-face design where each patient received both treatments on opposite sides of the face. Effectiveness was assessed using the NLF-SRS and GAIS, along with patient satisfaction surveys. Safety was monitored through AE reporting. The authors of the study aimed to determine whether PFL was not inferior to JUXC in terms of effectiveness and safety for NLF correction (Figures 4-6).

**Figure 7.**
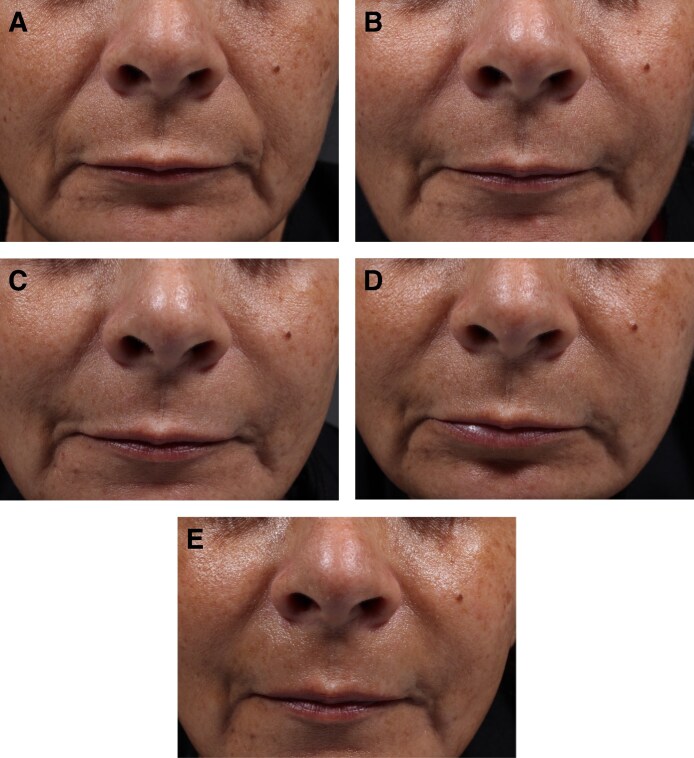
Before and after of a 53-year-old female patient treated with 1.0 cc of Juvéderm Ultra XC on the right side and 1.0 cc of Princess FILLER Lidocaine on the left side with symmetrically severe nasolabial folds at baseline (A), mild nasolabial folds after 12 (B), 24 (C), and 36 (D) weeks and symmetrically moderate nasolabial folds at Week 48 (E).

**Figure 8.**
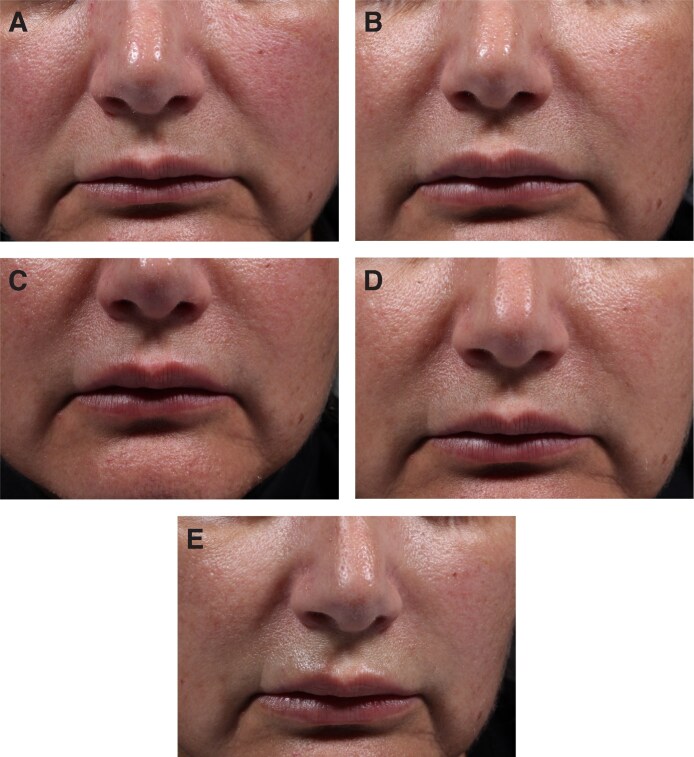
Before and after of a 49-year-old female patient treated with 0.9 cc of Juvéderm Ultra XC on the right side and with 1.0 cc of Princess FILLER Lidocaine on the left side with symmetrically severe nasolabial folds at baseline (A), mild nasolabial folds on the right and none on the left after 12 (B), and mild nasolabial folds on both sides after 24 (C), 36 weeks (D), and 48 weeks (E).

**Figure 9.**
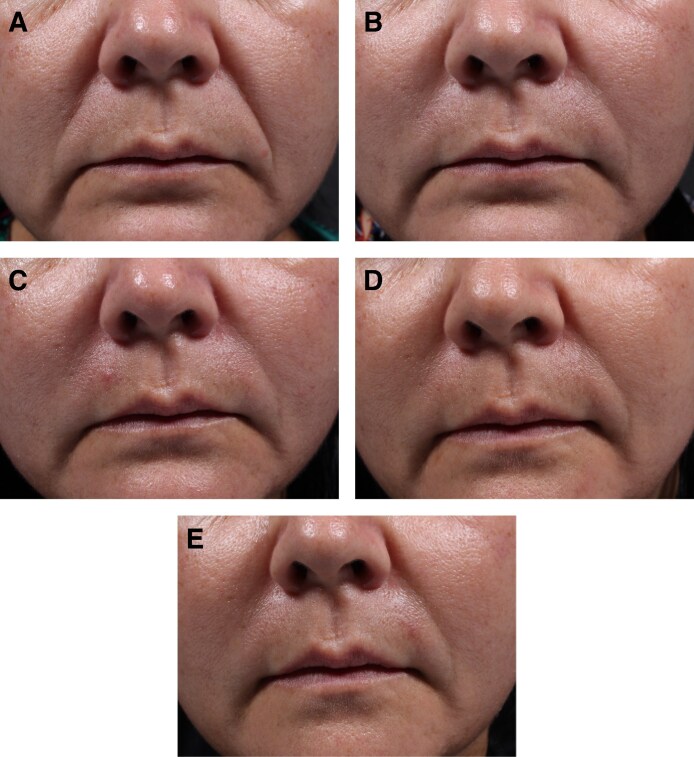
Before and after of a 47-year-old female patient treated with 1.0 cc of Princess FILLER Lidocaine on the right side and 0.8 cc of Juvéderm Ultra XC on the left side with symmetrically severe nasolabial folds at baseline (A), no nasolabial folds on both sides after 12 weeks (B), and mild nasolabial folds after 24 (C), 36 weeks (D) and 48 weeks (E).

The authors found that both PFL and JUXC were similarly effective in reducing the severity of NLFs, as measured by the NLF-SRS (Figures 7–9) and the GAIS. PFL demonstrated noninferiority to JUXC, with slightly higher percentages of patients achieving significant reductions in fold severity. Patient satisfaction, assessed through FACE-Q questionnaires, was comparable for both treatments. AEs were mostly mild or moderate and similar regarding frequency, indicating a comparable safety profile for PFL and JUXC in treating moderate-to-severe NLFs. PFL’s viscoelastic properties are key to its effectiveness. PFL has a *G*′ (elastic modulus) of ∼150,000 mPa (at 1 rad/s) and a *G*″ (viscous modulus) of ∼27,000 mPa. These values highlight a filler with much more elastic than viscous properties, enabling the filler to maintain its shape against the static forces resulting from structural changes in the maxilla and fat compartment displacement. These properties are further essential because, despite the NLF’s structural origins, there is significant movement in its proximity because of the cutaneous insertions of the perioral musculature.^2,3^ Such dynamic activity demands a filler that is not only able to correct volume deficits caused by bone and fat tissue changes but also resilient enough to accommodate the high amount of cutaneous movement without distortion. The moderate *G*′ value ensures that it is firm enough to provide volume where needed, still malleable enough to adapt to the subtleties of facial expressions, thus allowing the filler to deliver a natural-looking result. This happens through the smooth integration of the filler into the tissue, reducing the risk of visibility on the skin's surface, resulting in durable correction in the nasolabial area, thereby effectively mastering both the static and the dynamic challenges presented by the NLF.^13-15^ Addressing the dynamic challenges was also supported by the results of this study (Supplemental Table 2, available online at https://doi.org/10.1093/asj/sjaf137), namely the FACE-Q Subject Appraisal of NLFs, with 78.2% and 45.2% of the patients being bothered not at all or a little by how their NLFs look when they smile at Week 12 and Week 48, respectively, after treatment with PFL.

In addressing the complexities of NLF augmentation, a combination of injection techniques is often employed to achieve a natural and voluminous result. However, to establish safety and effectiveness profiles for each technique separately, a combination of different injection techniques, placing the product into the mid–to-deep dermis, was not allowed for the baseline treatment in this study. In a real-world setting, an ideal combination of different techniques might lead to even better results: the retrograde method involves inserting the full length of the needle into the fold or area of skin indentation and then releasing the filler in a controlled manner as the needle is carefully withdrawn. This approach allows for a precise linear deposition of the product along the deeper aspects of the fold. For the cranial depression near the NLF and the pyriform fossa, an enhanced retrograde technique is typically used, focusing on a greater product placement at the uppermost part of the fold to address the deeper areas of tissue loss. Transitioning to the middle and lower thirds of the NLF, a fanning technique can be utilized. This method starts similarly to the retrograde approach but with an added nuance; instead of completely removing the needle after the initial line of filler is placed, the direction of the needle is altered, and additional product is deposited along new lines. By repeating this process, the practitioner creates a fan-like pattern of filler distribution, which is ideal for treating larger surface areas and for sculpting the facial volume with a single-entry point. Utilizing this strategic combination of both retrograde and fan techniques allows for a layered and multidirectional fill, sculpting the nasolabial area with precision and creating a harmonious, youthful contour to the face. In the context of the retrograde technique, PFL's elasticity allows it to be deposited smoothly, providing immediate volume replenishment and support in the deeper layers of the fold, particularly in the cranial aspect in which bone resorption is more prominent. The moderate *G*′ ensures that the filler stays in place, resisting displacement from facial movements or gravity. Because the fan technique is applied to the NLF, the viscoelastic nature of the filler facilitates its spread in a fan-like pattern from a single injection point, achieving broader coverage without compromising the natural facial contours. The product’s properties prevent it from spreading too thin or migrating, which is essential for maintaining the desired and predictable aesthetic outcome over time.

Interestingly, the effectiveness of PFL and JUXC differed significantly when assessed by the independent blinded evaluator, performing a live assessment, and when assessed by the blinded evaluators assessing the photographs. A reason for this can be found in the differences between live assessment and photograph assessment. Photographs, even in 3-dimensional formats, fall short of conveying the true depth and dynamic contours of NLFs. They capture a static moment, lacking the ability to depict the folds in motion, which is critical given that the appearance of NLFs can significantly change with facial expressions. Furthermore, variables such as lighting and camera angle can distort the true severity of folds, casting shadows or creating highlights that misrepresent their actual depth. The resolution and quality of photographs are also subject to variability, which can obscure fine details essential for a nuanced evaluation. The subjective nature of interpreting 2-dimensional images can lead to inconsistencies in evaluation, because the inherent flatness of photographs impedes accurate judgment. Additionally, patient-to-patient variability poses another layer of complexity; individual anatomical differences, and the skin's interaction with light can vary significantly, making it challenging to standardize assessments across different patients.^16-18^ These limitations of photographic analysis are starkly highlighted when juxtaposed with the findings from live assessments, where evaluators, through direct interaction, were able to perceive the full extent of clinical improvement. The discrepancy in response rates—82.2% for PFL and 81.9% for JUXC based on live assessment, vs 61.1% for PFL and 64.1% for JUXC based on photographic review—underscores the importance of in-person evaluations. Live assessments capture the nuances of NLFs with greater accuracy, recognizing the intricate interplay between the treatment and the dynamic facial expressions, which is paramount for treatments in areas as complex as the nasolabial region.

PFL demonstrated an impressive 82.2% responder rate at Week 24, which is notably higher when compared with the results of Restylane and Restylane Lyft, as reported in a study by Li et al, in which the improvement rate—defined as a ≥1 point enhancement on the Wrinkle Severity Rating Scale (WSRS) after 24 weeks—was recorded at 64% and 65%, respectively.^19^ Furthermore, these figures surpass the responder rates observed in a study by Wu et al, which showed a 58.0% improvement with BioHyalux and a 63.8% improvement with Restylane 24 weeks after treatment of the NLF.^20^

A blinded and live evaluation by Li et al unveiled responder rates of 62.9% for Belotero Balance and 64.9% for Restylane Lyft in a recent publication.^21^ Although these findings are commendable, they are somewhat outrun by the outcomes associated with Vycross 17.5%. Monheit et al, in their studies, reported a remarkable 93.2% responder rate, following a similar blinded and live assessment protocol after 6 months.^22^ These rates were closely mirrored in a study involving the Chinese population by Yun et al, which reported an 84.2% response rate, which is very comparable with the effectiveness of PFL.^23^ In their study, Goodman et al presented Juvéderm Ultra Plus achieving a high response rate of 90%, however, in a nonblinded setting, suggesting that assessment conditions might significantly influence perceived effectiveness rates.

PFL's slightly lower response rate compared with Vycross 17.5% should not be directly attributed to the difference in the products themselves. Potential explanations for this variance could lie within the study settings. Factors such as demographic variations, assessment methodologies, and the scales used to measure improvements could all contribute to these differences. For example, the cultural perceptions of aesthetics, the severity of NLFs at baseline, and the evaluators’ training in using the WSRS might have influenced the outcomes.^12,24^ In the study performed by Monheit et al a median volume of 1.7 cc of VYC-17.5 was injected per NLF (initial and touch-up treatment), whereas a median volume of 1.4 cc of PFL was injected in this study. This might be a further explanation for the lower response rate, because 17.6% less of PFL was injected. One of the TEADEs reported in this study included 2 cases described as “eyelid margin crusting.” These events were recorded verbatim from the patients’ report and evaluated through follow-up clinical assessment. After thorough review and discussion with the treating investigators and medical monitor, it was determined that the events were not causally related to the treatment procedure or the study device. The symptom was transient, self-limiting, and resolved without the need for medical intervention. This assessment was also submitted to the appropriate regulatory authorities. Because no clinical signs of complication were observed and no diagnostic or photographic documentation was warranted at the time, the event was not further substantiated with imagery. This highlights the importance of careful attribution of AEs, especially when relying on subjective or patient-reported descriptors.

The clinical investigation was a methodically structured study, yet it presents certain limitations that are inherent in such research. The study's design was a randomized, patient-blinded and evaluating investigator-blinded, active treatment controlled, noninferiority, multicenter paired comparison, which, although robust, could be subject to biases, especially in assessments such as the NLF-SRS and the GAIS. Although measures were taken to mitigate these biases, such as blinded evaluators and randomized treatment allocations, the subjective nature of the scales could still introduce variability.

The multicenter aspect, involving 10 different sites, could lead to inconsistencies in application techniques, evaluation, and patient care, despite standardized protocols. The paired (split-face) comparison introduces a unique challenge as each side of the face serves as a control for the other, which could affect the objectivity of patient and investigator assessments because of potential side-to-side differences of skin characteristics that are not perfectly symmetrical.

The study population was limited to participants 22 years or older, but it did not specify a cap on age, which could lead to a wide range of skin types and responses to treatment.

The exclusion criteria were comprehensive, aiming to mitigate confounding factors, but they also limit the generalizability of the study's results to a broader population. Those with underlying conditions or previous treatments, which are common in cosmetic practice, were not represented. Furthermore, the study's registration at ClinicalTrials.gov and adherence to international and national laws ensure transparency and ethical conduct, but they do not necessarily account for all sources of bias that could affect the study's findings.

The timing of the effectiveness assessments and additional safety evaluations, scheduled at specific weeks, was well-structured, but they leave periods in which potential transient or delayed adverse effects may not be observed or documented. Moreover, the option for a repeat treatment at Week 36 or 48, depending on the patient’s condition, introduces another variable that could complicate the assessment of long-term effectiveness and safety.

The alterations in the study protocol, including the pause in repeat treatments and subsequent introduction of a new follow-up visit, present several potential limitations. The interruption in the treatment schedule could lead to inconsistencies in data, because the gap in treatment and evaluation might affect the continuity and comparability of results. This break in protocol could also impact the overall effectiveness of the treatment, because the timing of interventions is often critical in clinical studies. The inclusion of an additional follow-up (Visit 7c) in the retreatment phase may introduce response variability, which is attributable to the varied pause lengths in treatment or individual patient characteristics. It is crucial to note, however, that the results presented in this manuscript pertain exclusively to the initial treatment phase. The mention of Visit 7c's potential impact is to acknowledge its relevance in the retreatment context, ensuring comprehensive reporting within the study's scope. The resumption of treatments and evaluations might not adequately account for any changes in patients’ conditions during the pause. This could lead to an underestimation or overestimation of the treatment's effectiveness or safety. One limitation of this study is the lack of objective imaging data to confirm filler integration within the tissue. Although high patient satisfaction and natural appearance were captured through FACE-Q scores, these subjective measures do not directly evaluate the anatomical distribution or structural integration of the filler. Future studies incorporating ultrasound or other imaging modalities would be valuable to more fully characterize filler behavior and confirm integration beyond patient-reported outcomes.

## CONCLUSIONS

In this study, PFL has established itself as a noninferior treatment to JUXC for moderate-to-severe NLFs, demonstrating comparable effectiveness and safety. Both treatments achieved notable reductions in fold severity, with similar satisfaction ratings from patients. The slight edge of PFL in effectiveness is supported by its viscoelastic properties, tailored to address the static and dynamic complexities of NLFs. Comparable AE profiles further underscore the treatments’ parity. PFL's moderate *G*′ and tan *δ* values, indicating a balance of firmness and adaptability, are critical for a filler's performance in areas of frequent movement like the perioral region. The study's rigorous design, encompassing a split-face methodology across multiple centers, strengthens the validity of its findings.

## Supplemental Material

This article contains supplemental material located online at https://doi.org/10.1093/asj/sjaf137.

## Supplementary Material

sjaf137_Supplementary_Data
