# Supplementary material for: Multicenter, Randomized Split-Face Trial of a Crosslinked Hyaluronic Acid Fillers With Lidocaine for Nasolabial Fold Correction
Source: Aesthet Surg J. 2025 Aug 1;46(7):759–69. doi: 10.1093/asj/sjaf137 (PMC13268692; doi:10.1093/asj/sjaf137)
Supplement: sjaf137_Supplementary_Data [file sjaf137_supplementary_data.zip › Supplemental Table 1.docx]

**Supplemental Table 1**. Summary of Subjects Satisfaction with Overall Outcome of the Treatment Using the Face-Q Questionnaire Satisfaction with Outcome

|  | | Princess FILLER Lidocaine (N=270) _______________________________________________ Visit | | | | Juvederm Ultra XC (N=270) _______________________________________________ Visit | | | | |
| --- | --- | --- | --- | --- | --- | --- | --- | --- | --- | --- |
|  | Category | Week 12 n (%) | Week 24 n (%) | Week 36 n (%) | Week 48 n (%) | Week 12 n (%) | Week 24 n (%) | Week 36 n (%) | Week 48 n (%) |  |
| Pleased | Definitely disagree | 8 (3.0 %) | 13 (4.8 %) | 5 (1.9 %) | 7 (2.6 %) | 7 (2.6 %) | 13 (4.8 %) | 7 (2.6 %) | 5 (1.9 %) |  |
|  | Somewhat disagree | 7 (2.6 %) | 11 (4.1 %) | 10 (3.7 %) | 11 (4.1 %) | 11 (4.1 %) | 18 (6.7 %) | 12 (4.4 %) | 17 (6.3 %) |  |
|  | Somewhat agree | 64 (23.7 %) | 64 (23.7 %) | 74 (27.4 %) | 82 (30.4 %) | 63 (23.3 %) | 67 (24.8 %) | 70 (25.9 %) | 73 (27.0 %) |  |
|  | Definitely agree | 179 (66.3 %) | 161 (59.6 %) | 113 (41.9 %) | 111 (41.1 %) | 177 (65.6 %) | 151 (55.9 %) | 113 (41.9 %) | 116 (43.0 %) |  |
|  | (Missing) | 12 (4.4 %) | 21 (7.8 %) | 68 (25.2 %) | 59 (21.9 %) | 12 (4.4 %) | 21 (7.8 %) | 68 (25.2 %) | 59 (21.9 %) |  |
| Expected | Definitely disagree | 13 (4.8 %) | 16 (5.9 %) | 8 (3.0 %) | 10 (3.7 %) | 12 (4.4 %) | 16 (5.9 %) | 7 (2.6 %) | 8 (3.0 %) |  |
|  | Somewhat disagree | 26 (9.6 %) | 24 (8.9 %) | 19 (7.0 %) | 24 (8.9 %) | 25 (9.3 %) | 35 (13.0 %) | 21 (7.8 %) | 24 (8.9 %) |  |
|  | Somewhat agree | 77 (28.5 %) | 93 (34.4 %) | 89 (33.0 %) | 93 (34.4 %) | 87 (32.2 %) | 85 (31.5 %) | 89 (33.0 %) | 93 (34.4 %) |  |
|  | Definitely agree | 142 (52.6 %) | 116 (43.0 %) | 86 (31.9 %) | 84 (31.1 %) | 134 (49.6 %) | 113 (41.9 %) | 85 (31.5 %) | 86 (31.9 %) |  |
|  | (Missing) | 12 (4.4 %) | 21 (7.8 %) | 68 (25.2 %) | 59 (21.9 %) | 12 (4.4 %) | 21 (7.8 %) | 68 (25.2 %) | 59 (21.9 %) |  |
| Great | Definitely disagree | 11 (4.1 %) | 14 (5.2 %) | 7 (2.6 %) | 7 (2.6 %) | 9 (3.3 %) | 15 (5.6 %) | 10 (3.7 %) | 6 (2.2 %) |  |
|  | Somewhat disagree | 14 (5.2 %) | 18 (6.7 %) | 12 (4.4 %) | 22 (8.1 %) | 16 (5.9 %) | 28 (10.4 %) | 18 (6.7 %) | 23 (8.5 %) |  |
|  | Somewhat agree | 70 (25.9 %) | 73 (27.0 %) | 88 (32.6 %) | 83 (30.7 %) | 75 (27.8 %) | 74 (27.4 %) | 76 (28.1 %) | 86 (31.9 %) |  |
|  | Definitely agree | 163 (60.4 %) | 144 (53.3 %) | 95 (35.2 %) | 99 (36.7 %) | 158 (58.5 %) | 132 (48.9 %) | 98 (36.3 %) | 96 (35.6 %) |  |
|  | (Missing) | 12 (4.4 %) | 21 (7.8 %) | 68 (25.2 %) | 59 (21.9 %) | 12 (4.4 %) | 21 (7.8 %) | 68 (25.2 %) | 59 (21.9 %) |  |
|  | | Princess FILLER Lidocaine (N=270) _______________________________________________ Visit | | | | Juvederm Ultra XC (N=270) _______________________________________________ Visit | | | | |
|  | Category | Week 12 n (%) | Week 24 n (%) | Week 36 n (%) | Week 48 n (%) | Week 12 n (%) | Week 24 n (%) | Week 36 n (%) | Week 48 n (%) |  |
| Look in mirror | Definitely disagree | 12 (4.4 %) | 16 (5.9 %) | 12 (4.4 %) | 12 (4.4 %) | 12 (4.4 %) | 17 (6.3 %) | 11 (4.1 %) | 12 (4.4 %) |  |
|  | Somewhat disagree | 28 (10.4 %) | 32 (11.9 %) | 20 (7.4 %) | 35 (13.0 %) | 31 (11.5 %) | 41 (15.2 %) | 34 (12.6 %) | 38 (14.1 %) |  |
|  | Somewhat agree | 81 (30.0 %) | 89 (33.0 %) | 90 (33.3 %) | 88 (32.6 %) | 85 (31.5 %) | 86 (31.9 %) | 80 (29.6 %) | 90 (33.3 %) |  |
|  | Definitely agree | 137 (50.7 %) | 112 (41.5 %) | 80 (29.6 %) | 76 (28.1 %) | 130 (48.1 %) | 105 (38.9 %) | 77 (28.5 %) | 71 (26.3 %) |  |
|  | (Missing) | 12 (4.4 %) | 21 (7.8 %) | 68 (25.2 %) | 59 (21.9 %) | 12 (4.4 %) | 21 (7.8 %) | 68 (25.2 %) | 59 (21.9 %) |  |
| Fantastic | Definitely disagree | 17 (6.3 %) | 20 (7.4 %) | 14 (5.2 %) | 17 (6.3 %) | 18 (6.7 %) | 22 (8.1 %) | 17 (6.3 %) | 19 (7.0 %) |  |
|  | Somewhat disagree | 30 (11.1 %) | 41 (15.2 %) | 34 (12.6 %) | 41 (15.2 %) | 34 (12.6 %) | 42 (15.6 %) | 37 (13.7 %) | 37 (13.7 %) |  |
|  | Somewhat agree | 85 (31.5 %) | 86 (31.9 %) | 73 (27.0 %) | 81 (30.0 %) | 88 (32.6 %) | 86 (31.9 %) | 69 (25.6 %) | 88 (32.6 %) |  |
|  | Definitely agree | 126 (46.7 %) | 102 (37.8 %) | 81 (30.0 %) | 72 (26.7 %) | 118 (43.7 %) | 99 (36.7 %) | 79 (29.3 %) | 67 (24.8 %) |  |
|  | (Missing) | 12 (4.4 %) | 21 (7.8 %) | 68 (25.2 %) | 59 (21.9 %) | 12 (4.4 %) | 21 (7.8 %) | 68 (25.2 %) | 59 (21.9 %) |  |
| Miraculous | Definitely disagree | 30 (11.1 %) | 32 (11.9 %) | 21 (7.8 %) | 31 (11.5 %) | 32 (11.9 %) | 35 (13.0 %) | 29 (10.7 %) | 31 (11.5 %) |  |
|  | Somewhat disagree | 46 (17.0 %) | 38 (14.1 %) | 44 (16.3 %) | 43 (15.9 %) | 41 (15.2 %) | 45 (16.7 %) | 44 (16.3 %) | 42 (15.6 %) |  |
|  | Somewhat agree | 85 (31.5 %) | 105 (38.9 %) | 83 (30.7 %) | 91 (33.7 %) | 94 (34.8 %) | 97 (35.9 %) | 76 (28.1 %) | 96 (35.6 %) |  |
|  | Definitely agree | 97 (35.9 %) | 74 (27.4 %) | 54 (20.0 %) | 46 (17.0 %) | 91 (33.7 %) | 72 (26.7 %) | 53 (19.6 %) | 42 (15.6 %) |  |
|  | (Missing) | 12 (4.4 %) | 21 (7.8 %) | 68 (25.2 %) | 59 (21.9 %) | 12 (4.4 %) | 21 (7.8 %) | 68 (25.2 %) | 59 (21.9 %) |  |

N = number of subjects in corresponding population and subgroup; n = number of subjects in a given category. The percentage is based on the number of subjects in the corresponding population and subgroup.

(Full Analysis Set). FACE-Q^®^ is a U.S. registered trademark of Memorial Sloan-Kettering Cancer Center, 1275 York Avenue, New York, NY 10065. © 2013 Memorial Sloan-Kettering Cancer Center, Memorial Hospital for Cancer and Allied Diseases, Sloan-Kettering Institute for Cancer Research, Anne Klassen, and Stefan Cano. All rights reserved.
