# Supplementary material for: Multicenter, Randomized Split-Face Trial of a Crosslinked Hyaluronic Acid Fillers With Lidocaine for Nasolabial Fold Correction
Source: Aesthet Surg J. 2025 Aug 1;46(7):759–69. doi: 10.1093/asj/sjaf137 (PMC13268692; doi:10.1093/asj/sjaf137)
Supplement: sjaf137_Supplementary_Data [file sjaf137_supplementary_data.zip › Supplemental Table 2.docx]

**Supplemental Table 2**. Summary of Subject Appearance - Appraisal of Nasolabial Folds Using the Face-Q (Full Analysis Set)

|  | | Princess FILLER Lidocaine (N=270) _______________________________________________ Visit | | | | Juvederm Ultra XC (N=270) _______________________________________________ Visit | | | |
| --- | --- | --- | --- | --- | --- | --- | --- | --- | --- |
|  | Category | Week 12 n (%) | Week 24 n (%) | Week 36 n (%) | Week 48 n (%) | Week 12 n (%) | Week 24 n (%) | Week 36 n (%) | Week 48 n (%) |
| Deep | Not at all | 131 (48.5 %) | 93 (34.4 %) | 52 (19.3 %) | 52 (19.3 %) | 133 (49.3 %) | 91 (33.7 %) | 51 (18.9 %) | 52 (19.3 %) |
|  | A little | 77 (28.5 %) | 98 (36.3 %) | 79 (29.3 %) | 73 (27.0 %) | 75 (27.8 %) | 89 (33.0 %) | 71 (26.3 %) | 68 (25.2 %) |
|  | Moderately | 38 (14.1 %) | 47 (17.4 %) | 58 (21.5 %) | 71 (26.3 %) | 37 (13.7 %) | 55 (20.4 %) | 66 (24.4 %) | 80 (29.6 %) |
|  | Extremely | 12 (4.4 %) | 12 (4.4 %) | 13 (4.8 %) | 15 (5.6 %) | 13 (4.8 %) | 15 (5.6 %) | 14 (5.2 %) | 11 (4.1 %) |
|  | (Missing) | 12 (4.4 %) | 20 (7.4 %) | 68 (25.2 %) | 59 (21.9 %) | 12 (4.4 %) | 20 (7.4 %) | 68 (25.2 %) | 59 (21.9 %) |
| Relaxed | Not at all | 142 (52.6 %) | 106 (39.3 %) | 56 (20.7 %) | 58 (21.5 %) | 145 (53.7 %) | 107 (39.6 %) | 52 (19.3 %) | 61 (22.6 %) |
|  | A little | 71 (26.3 %) | 95 (35.2 %) | 84 (31.1 %) | 65 (24.1 %) | 69 (25.6 %) | 76 (28.1 %) | 77 (28.5 %) | 60 (22.2 %) |
|  | Moderately | 34 (12.6 %) | 40 (14.8 %) | 48 (17.8 %) | 73 (27.0 %) | 35 (13.0 %) | 59 (21.9 %) | 57 (21.1 %) | 77 (28.5 %) |
|  | Extremely | 11 (4.1 %) | 9 (3.3 %) | 14 (5.2 %) | 15 (5.6 %) | 9 (3.3 %) | 8 (3.0 %) | 16 (5.9 %) | 13 (4.8 %) |
|  | (Missing) | 12 (4.4 %) | 20 (7.4 %) | 68 (25.2 %) | 59 (21.9 %) | 12 (4.4 %) | 20 (7.4 %) | 68 (25.2 %) | 59 (21.9 %) |
| Old | Not at all | 128 (47.4 %) | 98 (36.3 %) | 62 (23.0 %) | 51 (18.9 %) | 126 (46.7 %) | 100 (37.0 %) | 55 (20.4 %) | 53 (19.6 %) |
|  | A little | 77 (28.5 %) | 89 (33.0 %) | 66 (24.4 %) | 75 (27.8 %) | 81 (30.0 %) | 83 (30.7 %) | 63 (23.3 %) | 69 (25.6 %) |
|  | Moderately | 38 (14.1 %) | 45 (16.7 %) | 57 (21.1 %) | 69 (25.6 %) | 39 (14.4 %) | 50 (18.5 %) | 67 (24.8 %) | 75 (27.8 %) |
|  | Extremely | 15 (5.6 %) | 18 (6.7 %) | 17 (6.3 %) | 16 (5.9 %) | 12 (4.4 %) | 17 (6.3 %) | 17 (6.3 %) | 14 (5.2 %) |
|  | (Missing) | 12 (4.4 %) | 20 (7.4 %) | 68 (25.2 %) | 59 (21.9 %) | 12 (4.4 %) | 20 (7.4 %) | 68 (25.2 %) | 59 (21.9 %) |
|  | | Princess FILLER Lidocaine (N=270) _______________________________________________ Visit | | | | Juvederm Ultra XC (N=270) _______________________________________________ Visit | | | |
|  | Category | Week 12 n (%) | Week 24 n (%) | Week 36 n (%) | Week 48 n (%) | Week 12 n (%) | Week 24 n (%) | Week 36 n (%) | Week 48 n (%) |
| Smile | Not at all | 143 (53.0 %) | 119 (44.1 %) | 75 (27.8 %) | 55 (20.4 %) | 136 (50.4 %) | 118 (43.7 %) | 71 (26.3 %) | 56 (20.7 %) |
|  | A little | 68 (25.2 %) | 79 (29.3 %) | 62 (23.0 %) | 67 (24.8 %) | 78 (28.9 %) | 74 (27.4 %) | 57 (21.1 %) | 70 (25.9 %) |
|  | Moderately | 37 (13.7 %) | 43 (15.9 %) | 55 (20.4 %) | 74 (27.4 %) | 33 (12.2 %) | 46 (17.0 %) | 60 (22.2 %) | 71 (26.3 %) |
|  | Extremely | 10 (3.7 %) | 9 (3.3 %) | 10 (3.7 %) | 15 (5.6 %) | 11 (4.1 %) | 12 (4.4 %) | 14 (5.2 %) | 14 (5.2 %) |
|  | (Missing) | 12 (4.4 %) | 20 (7.4 %) | 68 (25.2 %) | 59 (21.9 %) | 12 (4.4 %) | 20 (7.4 %) | 68 (25.2 %) | 59 (21.9 %) |
| Compared | Not at all | 160 (59.3 %) | 123 (45.6 %) | 71 (26.3 %) | 67 (24.8 %) | 153 (56.7 %) | 119 (44.1 %) | 72 (26.7 %) | 66 (24.4 %) |
|  | A little | 50 (18.5 %) | 72 (26.7 %) | 70 (25.9 %) | 65 (24.1 %) | 60 (22.2 %) | 67 (24.8 %) | 64 (23.7 %) | 64 (23.7 %) |
|  | Moderately | 37 (13.7 %) | 45 (16.7 %) | 44 (16.3 %) | 67 (24.8 %) | 33 (12.2 %) | 52 (19.3 %) | 50 (18.5 %) | 71 (26.3 %) |
|  | Extremely | 11 (4.1 %) | 10 (3.7 %) | 17 (6.3 %) | 12 (4.4 %) | 12 (4.4 %) | 12 (4.4 %) | 16 (5.9 %) | 10 (3.7 %) |
|  | (Missing) | 12 (4.4 %) | 20 (7.4 %) | 68 (25.2 %) | 59 (21.9 %) | 12 (4.4 %) | 20 (7.4 %) | 68 (25.2 %) | 59 (21.9 %) |

N = number of subjects in corresponding population and subgroup; n = number of subjects in a given category. The percentage is based on the number of subjects in the corresponding population and subgroup.

FACE-Q^®^ is a U.S. registered trademark of Memorial Sloan-Kettering Cancer Center, 1275 York Avenue, New York, NY 10065. © 2013 Memorial Sloan-Kettering Cancer Center, Memorial Hospital for Cancer and Allied Diseases, Sloan-Kettering Institute for Cancer Research, Anne Klassen, and Stefan Cano. All rights reserved.
